# Pathway-GPS and SIGORA: identifying relevant pathways based on the over-representation of their gene-pair signatures

**DOI:** 10.7717/peerj.229

**Published:** 2013-12-19

**Authors:** Amir B.K. Foroushani, Fiona S.L. Brinkman, David J. Lynn

**Affiliations:** 1 Animal & Bioscience Research Department, AGRIC, Teagasc, Grange, Dunsany, Co. Meath, Ireland; 2 Department of Molecular Biology and Biochemistry, Simon Fraser University, Burnaby, British Columbia, Canada

**Keywords:** Systems biology, Functional analysis, Over-representation analysis, Pathway analysis, Shared components, High-throughput data

## Abstract

**Motivation.** Predominant pathway analysis approaches treat pathways as collections of individual genes and consider all pathway members as equally informative. As a result, at times spurious and misleading pathways are inappropriately identified as statistically significant, solely due to components that they share with the more relevant pathways.

**Results.** We introduce the concept of Pathway Gene-Pair Signatures (Pathway-GPS) as pairs of genes that, as a combination, are specific to a single pathway. We devised and implemented a novel approach to pathway analysis, Signature Over-representation Analysis (SIGORA), which focuses on the statistically significant enrichment of Pathway-GPS in a user-specified gene list of interest. In a comparative evaluation of several published datasets, SIGORA outperformed traditional methods by delivering biologically more plausible and relevant results.

**Availability.** An efficient implementation of SIGORA, as an R package with precompiled GPS data for several human and mouse pathway repositories is available for download from http://sigora.googlecode.com/svn/.

## Introduction

Pathway analysis identifies biological pathways that are statistically enriched in a given dataset and plays a crucial role in the interpretation of high-throughput experimental datasets including gene or protein expression profiles (Khatri & Drăghici, 2005; Huang, Sherman & Lempicki, 2009) and genome-wide association studies (GWAS) (Wang, Li & Hakonarson, 2010). Pathway analysis can guide the understanding of complex biological datasets through the statistical association of observations at the molecular level to processes at the systems level. Such analysis can, for example, highlight processes that are dysregulated in certain pathological conditions, such as cancer (Copeland & Jenkins, 2009) or infection (Wherry et al., 2007).

Currently, two types of pathway analysis methods are widely used: Over-representation Analysis (ORA) methods (reviewed in Khatri & Drăghici, 2005) and methods related to Gene Set Enrichment Analysis (GSEA) (Mootha et al., 2003; Subramanian et al., 2005; Dinu et al., 2009).

Despite major differences between ORA and GSEA methods (see e.g., Emmert-Streib & Glazko, 2011, for a discussion), these approaches share a notable limitation: most current methods treat all genes in a given pathway as equal indicators that that pathway is significant. This assumption, that each gene in a pathway has the same power to distinguish one pathway from another, and that genes assume their roles without consideration of the context and expression of other genes, is undoubtedly flawed (Gillis & Pavlidis, 2011; Ma, Sartor & Jagadish, 2011; Khatri, Sirota & Butte, 2012).

**Figure 1:**
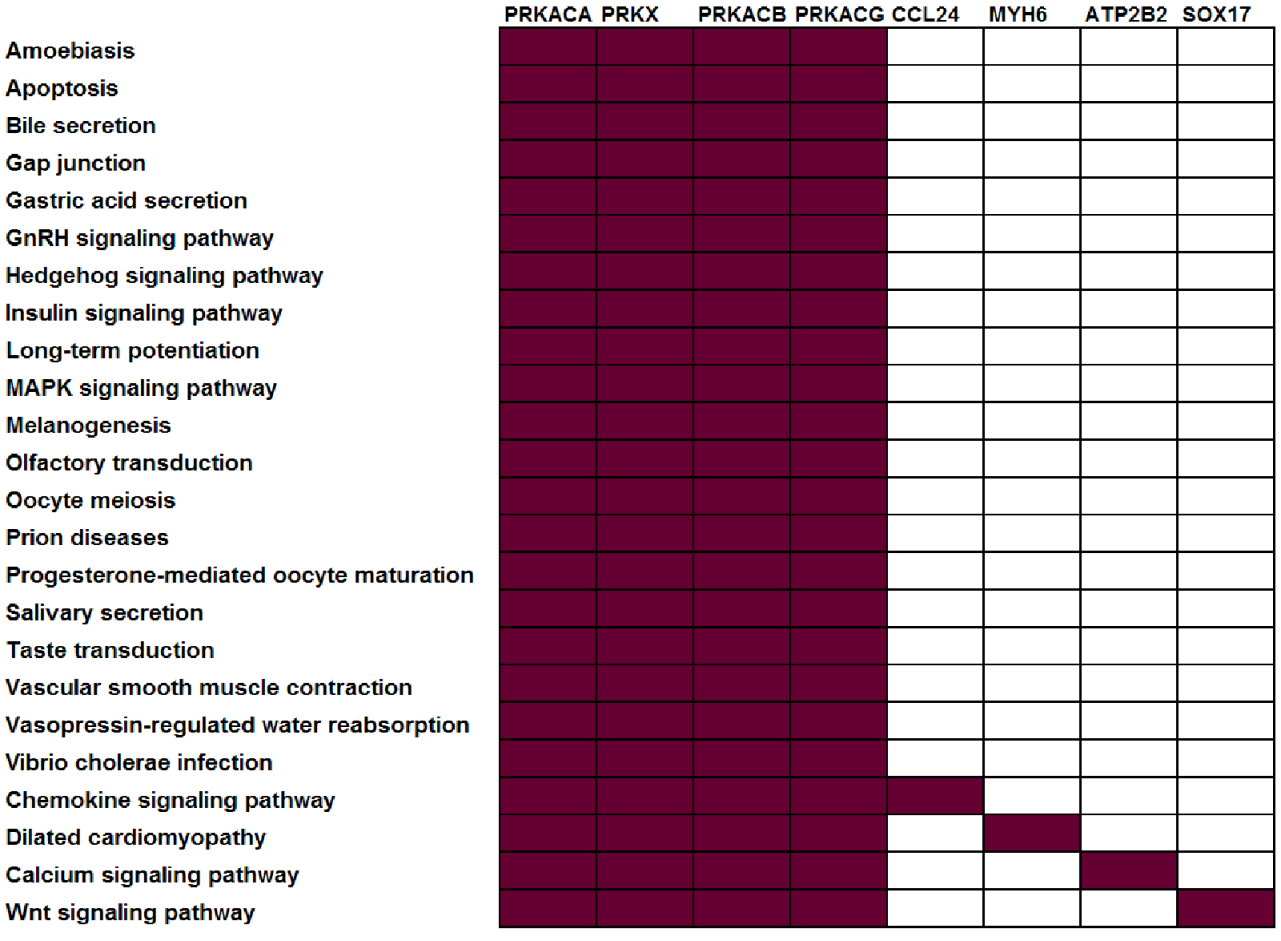
Not all genes have the same power to distinguish between different pathways. In this example, all current KEGG annotations of seven selected genes are shown. Red: annotated in pathway; white: not annotated in this pathway.

To illustrate this point, consider four protein kinases, PRKACA, PRKACB, PRKACG, and PRKX. Within the KEGG (Kanehisa et al., 2011) pathway repository, these genes are members of 24 different pathways, i.e., they co-occur in roughly 10% of KEGG human pathways (Fig. 1). Consider a dataset where all four of these genes were observed to be differentially expressed — many pathway analysis tools would identify all 24 different pathways as statistically significant leaving the biologist perplexed as to which of these pathways are the most biologically relevant to their study. The underlying problem (that genes may be associated with multiple pathways and, as such, that all genes are not equivalent “Signatures” of a given pathway) is widespread and not limited to kinases. Within KEGG, 52% of genes are annotated in more than one pathway (Table 1).

**Table 1:** Annotation and co-annotation of human genes in current pathway repositories. The number of human genes annotated in pathways in the KEGG, Reactome and PID databases. On average more than 40% of genes are annotated in more than one pathway whereas gene-pairs rarely co-occur in multiple pathways.

Pathway database	Number of annotated genes	% of genes annotated in a single pathway	% of genes annotated in multiple pathways	Number of co-annotated gene-pairs	% of gene-pairs that co-occur in a single pathway
KEGG	5,660	48%	52%	1,205,807	90%
REACTOME	5,046	62%	38%	197,034	87%
PID_BIOCARTA	1,368	54%	46%	32,361	78%
PID_NCI	2,374	51%	49%	116,852	87%

As a result, many pathway analysis methods return misleading statistically significant pathways that are significant solely due to shared components with other pathways (e.g., “*Prion Disease”* is identified as a significant pathway in a dengue fever microarray study (Hoang et al., 2010) simply because many of the genes annotated in this “pathway” are co-annotated in inflammation-related pathways).

Here, we report a novel approach to address this problem, which involves the identification of statistically over-represented Pathway Gene-Pair Signatures (Pathway-GPS) (i.e., weighted pairs of genes which uniquely occur together in a single pathway). The use of such gene pairs is also motivated by the data in Table 1: in contrast to single genes, co-annotated gene pairs tend to be specific to a single pathway. We provide an implementation of this approach in R (SIGORA; downloadable from http://sigora.googlecode.com/svn/). We describe this approach and demonstrate how SIGORA significantly reduces the identification of spurious pathways in analyses of simulated and real biological datasets.

## Materials and Methods

### Algorithm

**Figure 2:**
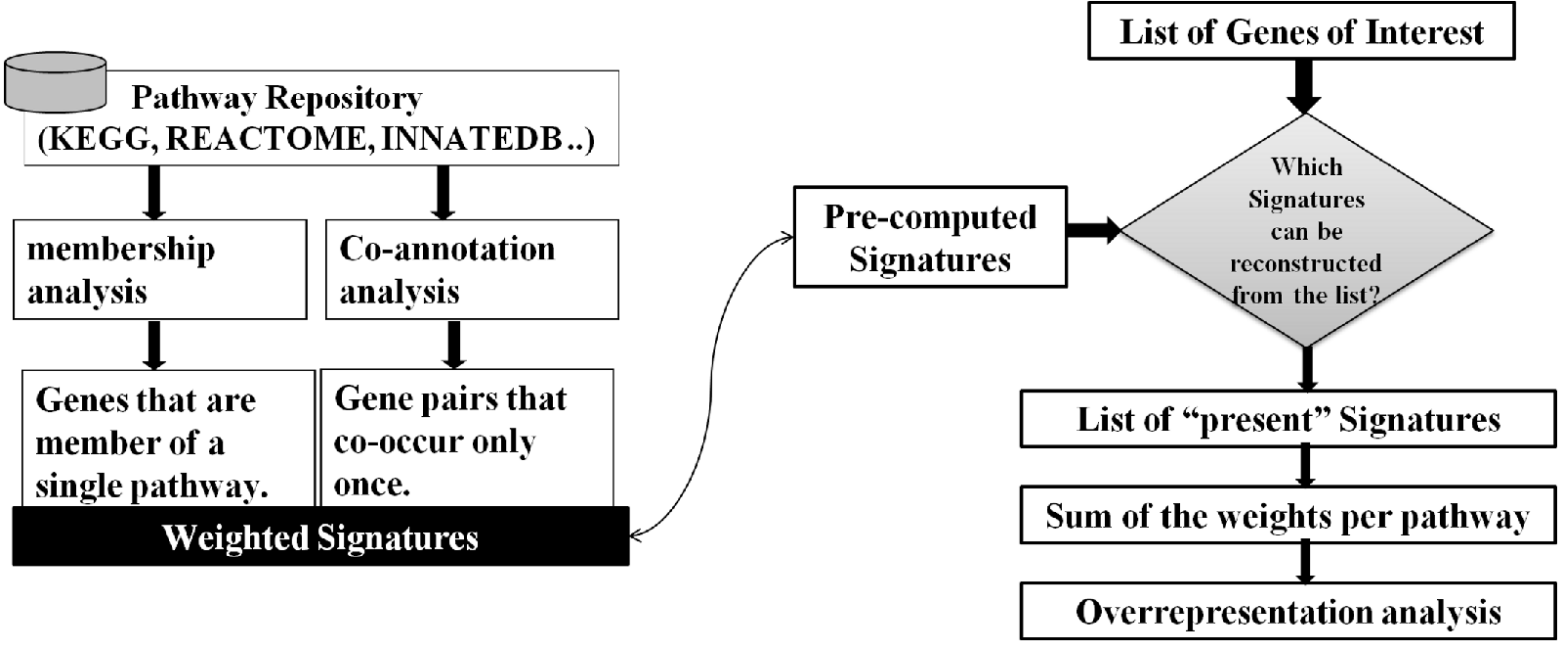
SIGORA’s two phases. In the off-line phase (left) a pathway repository is transformed to disjoint sets of weighted GPS. These precompiled signatures are used in the on-line phase (right) to evaluate a user specific input gene list.

As illustrated in Fig. 2, our approach to the problem consists of two phases: In an offline phase, we compile a set of weighted markers/Signatures for each pathway in a repository, which we call Pathway Gene-Pair Signatures (*Pathway-GPS*). Subsequently, in an online phase, the method identifies the statistical over-representation of such Signatures in a user-specified gene list using an adapted version of the hypergeometric test.

Given a pathway repository (e.g., KEGG), for each gene-pair in a pathway, SIGORA investigates the co-appearance of the two genes in other pathways of the repository. A gene-pair that uniquely occurs in a single pathway is considered a Signature of that pathway and is assigned a weight. The weight of a Signature (from [0, 1]) quantifies the average commitment of the components of the GPS towards the common pathway, i.e., the weight scores the reliability of the Signature as evidence for the associated pathway. For hierarchically organized repositories (like REACTOME (Matthews et al., 2009)), this process is repeated iteratively after the removal of pathways on the top level of the repository, i.e., in each iteration, new weighted Signatures are identified for the pathways on the lower, more specific levels of the hierarchy. Once this offline stage is completed, the resulting sets of weighted gene-pairs that represent each pathway are non-overlapping and can be *re-used* for pathway analysis of any user-specified gene lists.

When presented with a gene list of interest (e.g., genes that are differentially expressed), SIGORA determines which of the pairs from its (pre-compiled) Signature repository can be reconstructed from the genes in the list. A Signature is considered “present” only if both of its constituent genes are found in the user-specified query list. This inherently leads to the selection of the more relevant roles of a gene in the experimental context, as SIGORA relies on the status of the other genes in the pathway for the reconstruction of the Signatures. For each pathway, the weights of present Signatures are summed up and hypergeometric probabilities are used to assess the statistical significance of the observed Signature sets.

### Pathway Gene-Pair Signatures (Pathway-GPS)

**Figure 3:**
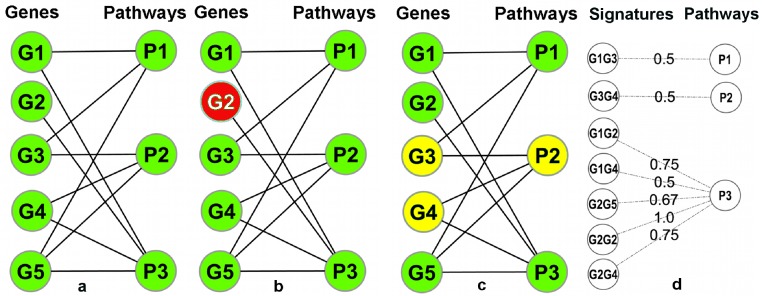
Overview of the signature transformation. (A) A schematic pathway repository as a bipartite graph (B); G1…G5: genes; P1…P3: pathways. (B) A pathway unique gene. (C) A Gene-Pair Signature (GPS): G3 and G4 co-occur only in P2. (D) Each GPS is associated with a single pathway and has a weight equal to the average inverse degree (in B) of its constituent genes.

A pathway database/repository contains (at least) two types of entities: pathways and genes. This can be represented by a bipartite graph (or bipartite network) *B* = (*V g*, *V p*, *E*) with two distinct sets of nodes (*Vg*: gene nodes and *Vp*: pathway nodes) where the edges in *E* connect the genes to the pathways and signify the annotation of a gene in a particular pathway (Fig. 3A). In this graph, the degrees (number of incident edges) of the pathway nodes correspond to pathway sizes (i.e., the number of genes annotated in the pathway) and the degree of the gene nodes corresponds to the number of different pathways a gene is annotated in. In particular, genes with degree one are exclusively annotated within a single pathway (‘*Pathway Unique Genes (PUGs)’*).

Using the igraph (Csardi & Nepusz, 2006) package of the R-Statistical Framework (R Development Core Team, 2008), *B* can be manipulated and transformed. A weighted one-mode projection (Newman, 2001) of *B* into the gene dimension, yields a new graph *Proj* = (*V g*, *E*′, *W*) with only one type of nodes (only gene nodes *Vg*), where two genes are connected by an edge in *E*′ if (and only if) they are co-annotated in one or more pathways, and each edge is associated with a weight (in *W*) which signifies the number of pathways that are shared between the two incident genes (i.e., the two genes connected by the edge). Two genes connected by an edge of weight one in *Proj* are, *as a combination,* unique to a single pathway. We call such pairs Pathway Gene-Pair Signatures (*Pathway-GPS*) of that pathway. The process of identifying PUGs and Pathway-GPS for all pathways in a given repository is termed the *‘Signature Transformation’* of the repository.

Hypothetically, one could go beyond gene-pairs and also consider *n*-tuples (with *n* > 2) of genes that co-occur in a single pathway *P* as signatures of *P*. The possible benefits of such extensions, however, do not currently seem to justify the associated complications to the computational and methodological framework. This point is further discussed in the Text S1.

### Assignment of weights to GPS

As, by definition, each Pathway-GPS is uniquely associated with a single pathway, identifying such Signatures in a gene list of interest (e.g., observing that both constituent genes of a Pathway-GPS are in the list of differentially expressed genes) can serve as an indicator of the activation/perturbation of the associated pathway. Each pathway can (and usually does) have multiple possible Pathway-GPS, and (as discussed below) the method does not rely on the observation of an individual GPS but rather on the statistical over-representation of multiple GPS in comparison to the expected proportion.

Yet before the over-representation of GPS can be used for the identification of relevant pathways, we need to emphasize that different GPS vary in their reliability as indicators of a particular pathway. This is due to the fact that, while the two genes comprising a Pathway-GPS can only be co-annotated in a single pathway, each of the two genes – *considered individually* – can be a member of several distinct pathways.

Consider GPS of the form (*g*1, *g*2) for a pathway *P*, where *g1* is annotated in *i* pathways and *g2* is in *j* pathways. Intuitively, a GPS that consists of two PUGs (i.e., a case where *i* = *j* = 1) is a more appealing signature than a GPS that consists of two ‘multifunctional’ genes (say, *i* = 4 and *j* = 3), where the simultaneous observation of the two genes might be due to other factors (e.g., simultaneous activation of two different pathways).

To address this issue, a weight is assigned to each GPS to quantify its reliability as an indicator of its associated pathway. Let (*g1, g2*) be a GPS associated with a pathway *P* and let *i* and *j* be the number of individual pathways annotations of *g1* and *g2*, respectively. The weight of the GPS is

\begin{eqnarray*} {w}_{i,j}=\frac{1}{2}\left(\frac{1}{i}+\frac{1}{j}\right)=\frac{i+j}{2\ast i\ast j}. \end{eqnarray*}

A motivation for this weighting scheme and a discussion of alternative weighting strategies can be found in the Text S1. In the implementation, the user also has access to several alternative weighting schemes and user defined schemes are also supported. Figure 3 summarizes the main ideas behind the compilation of weighted Signatures for a given pathway repository.

### Identifying statistically over-represented Pathway-GPS

Analogous to traditional (individual gene) ORA (IG-ORA) methods, the distribution function of hypergeometric probabilities is used to calculate *p*-values indicating the statistical enrichment of Pathway-GPS in a user-specified gene list, which is given by:

\begin{eqnarray*} \displaystyle p(k,n,m,N)=p(x\geq k)=1-\sum _{x=0}^{k-1}\frac{\left(\begin{array}{@{}c@{}} \displaystyle m\\ \displaystyle x \end{array}\right)\left(\begin{array}{@{}c@{}} \displaystyle N-m\\ \displaystyle n-x \end{array}\right)}{\left(\begin{array}{@{}c@{}} \displaystyle N\\ \displaystyle n \end{array}\right)}.&&\displaystyle \end{eqnarray*}

In individual gene ORA, *k* denotes the number of query genes in the tested pathway, *n* the number of the genes in the pathway, *N* the number of all (assayed or annotated) genes, and *m* the length of the query list (i.e., the number of the genes with interesting status). In contrast to these traditional approaches, however, the parameters of the hypergeometric function in SIGORA are calculated as sums of GPS weights rather than frequency statistics of individual gene annotations. The Signature ORA parameters for *p*-value calculations are summarized in Table 2.

**Table 2:** Interpretation of the hypergeometric distribution test parameters used in SIGORA. A GPS is **present** if *both* of its component genes are in the query list.

Parameter	Interpretation in SIGORA
*k* (success)	Rounded sum of the weights of all *present* GPS of the tested pathway
*n* (success states)	Rounded sum of the weights of all possible GPS of the tested pathway
*N* (universe)	Rounded sum of the weights of all possible GPS of the repository
*m* (sample size)	Rounded sum of the weights of all *present* GPS

Note that – *strictly speaking* – hypergeometric probabilities are only defined on natural numbers while the sums of GPS weights are positive floating point values, which is why the table refers to *rounded* values of the sums (to the closest integer). Theoretically, this rounding could lead to a *‘blurring of the weights for pathways with few GPS’*, however, in practice this issue hardly materializes, as such pathways are very rare (e.g., 219 out of 226 KEGG human pathways in our repository are associated with 10 or more possible GPS). In tests with simulated and real biological data sets, different choices of the rounding strategy (*floor*, *ceiling* or *nearest integer*) did not substantially affect the significance or the rank order of the identified pathways. As an aside, in the widely popular statistical framework R, the *phyper* function (which computes the distribution function of the hypergeometric distribution) does accept non-integer (floating point) parameters and handles such input by applying the following rounding strategy: the number of successes is rounded down, all remaining parameters are rounded to the closest integer value.

In our implementation, the user has the option to restrict the GPS-sets for the universe (*N*), and the success states (*n*) by providing a list of assayed genes (background). Furthermore, all PUGs are by default considered to represent a GPS of weight 1 (as a combination of the PUG with itself), but the user has the option to restrict the analysis to pairs of genuinely distinct genes.

### Multiple testing correction

Undertaking pathway analysis generally involves a large numbers of significance tests. As testing a multitude of hypotheses will inevitably lead to *some* ‘significant’ results, adjustment of *p*-values for multiple testing is a crucial feature of any pathway analysis tool. Bonferroni’s method is used by default in SIGORA for multiple testing correction (MTC). It is, however, relatively easy to change the MTC procedure, if a user prefers to explore other adjustment methods (see the implementation section).

### Selection of cut-off threshold for statistical significance of pathways

The choice of a reasonable cut-off threshold for statistical significance of the hypergeometric test results is an open methodological question in IG-ORA. In practice, values smaller than 0.1 or 0.05 after correction for multiple testing are commonly considered significant. Shifting the perspective from individual genes to the weighted Gene-Pair Signatures brings an additional challenge: as the size of the universe for the hypergeometric test dramatically increases (we move from a few thousand genes to up to a few hundred thousand weighted gene-pairs), the calculated *p*-values become by several orders of magnitude smaller than those observed in a typical IG-ORA analysis.

Based on our experience with simulated and biological datasets, we recommend a significance threshold of 0.001 after MTC (by Bonferroni). In the implementation, the default output of the analysis is the ranked list of pathways that achieve a corrected *p*-value up to this value. The user can also export the entire results table (including the *p*-values, corrected *p*-values and the parameters of the hypergeometric test) and the evidence (lists of present PUGs, list of the genes involved in present GPS or list of all present GPS along with their weights).

### Dealing with redundancies of semantic origin

Thus far, we have described the motivation for Signature Transformation from a *biological* perspective. However, in some cases, there are additional *semantic* reasons for sharing of components among pathways. In particular, some repositories (e.g., REACTOME (Matthews et al., 2009), INOH (Yamamoto et al., 2011) and Gene Ontology (Ashburner et al., 2000)) are organized in a hierarchical structure, where all genes associated with one pathway (child) are also included in a more general pathway (parent). This poses a general challenge for pathway analysis tools that ideally should identify the most relevant level of the hierarchy (Alexa, Rahnenführer & Lengauer, 2006; Grossmann et al., 2007; Jupiter, Sahutoglu & VanBuren, 2009).

The hierarchical nature of such repositories poses a special challenge for our method. As any gene-pairs from a child category also co-occur in the parent pathway, they would thus be excluded from being identified as a possible *Signature*. This would have the undesirable effect that all child pathways on the lower levels of the hierarchy would be left without any Signatures at all in the offline stage and hence be undetectable by SIGORA in the online stage.

To address this issue, we deploy the following iterative top-down strategy in the offline stage (Signature Transformation):

1. Set level = 1. Compile the repository Signatures as described for the non-hierarchical case. Assign the compiled signatures to level 1.
2. Remove all pathways from the top level of the hierarchy, increase the level and recompile Signatures for the remaining pathways. Assign the GPS to the current level. Iterate this step until no further hierarchical levels can be removed.

Figure S1 illustrates this procedure on a simplified hierarchical repository. In the *online* stage (the identification of significantly over-represented *Signatures* in a user-specified gene list) the user can specify how many levels of the hierarchy should be considered in the analysis. Any *GPS* (and any pathways) that are deeper down the hierarchy (i.e., are at a higher threshold level) are left out of the universe (and the analysis). If the user (for instance) asks for analysis up to the second level, then only *GPS* from levels one and two are considered.

The effect of this simple modification (i.e., the iterative strategy for Signature Transformation) is similar to a combination of the *Elim* (Alexa, Rahnenführer & Lengauer, 2006), and *TreeHugger* (Jupiter, Sahutoglu & VanBuren, 2009) algorithms in dealing with GO’s structure. *Elim* essentially excludes genes in more specific categories from consideration in more general categories, whereas *TreeHugger* weakens the contribution of such genes to the higher levels.

### Complexity and computational cost of the method

The complexity of the transformation step (in an implementation based on the outlined bipartite network interpretation of gene-pathway membership) is dominated by the complexity of the bipartite projection, which is given as:

\begin{eqnarray*} O(\Vert V\Vert \ast {d}^{2}+\Vert E\Vert ) \end{eqnarray*}

With ‖*V*‖: number of nodes in the network (number of genes + number of pathways), *d* the average degree of nodes, ‖*E*‖ the number of edges in the bipartite network.

For most inputs, the online phase (i.e., analysis of user specific input lists using the precompiled Signatures) completes within 15 s on a standard laptop (1.8 GHZ, 2 GB of RAM).

### Implementation

SIGORA is implemented as an R package. The package and a detailed manual are available for download from http://sigora.googlecode.com/svn/. The following highlights the most important steps in a typical work-follow in R (requires R version ≥2.10):


## install and load the downloaded package 
>install.packages(‘sigora_0.9.8.tar.gz’, type=‘source’, repos = NULL) 
>library(‘sigora’) 
## (please note that all of the following commands require that the package is already loaded) 
## import the query list from a file (assuming the list is given as Ensembl gene IDs): 
>myquerylist <-ens_converter(scan(‘myfile.txt’,what=‘character’)) 
## Alternatively, if the file consists of Entrez gene IDs 
>myquerylist <-entrez_converter(scan(‘myfile.txt’)) 
## perform signature over-representation analysis, using KEGG GPS 
>sigs(myquerylist,‘k’,markers=1,level=2) 
## multiple testing correction is done by Bonferroni and FDRs are also provided. In order to add Hommel’s method: 
>cbind(summary_results,p.adjust(summary_results[,5],‘hommel’)) 
## export the results into a file 
>export_results(filename=‘my_results.csv’, genes=T) 
## help (on Windows systems, help is shown in the web-browser) 
>help(sigs) 
## the following demo is also available 
>demo(sigora)


### Evaluation methods

We evaluated the performance of SIGORA by comparison to several other analysis tools on simulated and published biological datasets. Three of the methods compared are based on individual gene over-representation (*DAVID* (Huang, Sherman & Lempicki, 2009; Jiao et al., 2012), gProfileR (Reimand et al., 2007), *InnateDB* (Lynn et al., 2008)) and the remaining three (*GSEA_Preranked*, *GSEA* (Subramanian et al., 2005), *GSEA_AF* (Ma, Sartor & Jagadish, 2011)) are GSEA based. Acknowledging the inherent challenges associated with comparison of *p*-values across different statistical frameworks, for each tool we follow the recommendations of the authors of that tool regarding the choice of the most appropriate significance threshold and multiple testing correction (MTC) method (Text S1).

Among the methods listed above, we use *DAVID, gProfileR, GSEA_PRERANKED* for the simulation study, as these tools (like *SIGORA*) can be run on any pre-selected gene list and in ‘batch mode’. *GSEA* and Appearance frequency modulated GSEA (*AF*) are limited to particular experimental designs and have specific input data format requirements, and are used here only in the analysis of three biological dataset for which data in the required input format was available. *InnateDB* is used as a reference point in evaluation of the biological datasets, because SIGORA’s GPS are based on pathway annotation data as present in InnateDB. The rationale for selecting *GSEA* is its popularity; while *AF* was chosen because it attempts to address similar issues as SIGORA.

A short summary of the relevant characteristics of each of these methods is given below.

#### InnateDB (www.innatedb.com (Lynn et al., 2008; Breuer et al., 2013))

InnateDB’s pathway analysis interface provides traditional IG-ORA using the standard hypergeometric test. Its recommended MTC method is Benjamini–Hochberg. The pathway GPS in SIGORA’s current implementation are calculated using the pathway annotation as present in the latest release of InnateDB. In other words, any observed differences in analysis results between SIGORA and InnateDB are solely due to the differences between Signature-over-representation and individual-gene over-representation, and there are no additional confounding issues regarding the gene identifier mapping or different update status of the repositories across tools. We compare InnateDB to SIGORA using three biological datasets.

#### gProfileR (http://biit.cs.ut.ee/gprofiler/(Reimand et al., 2007))

gProfileR is the R package associated with the web-server of same name. Like InnateDB, it deploys a traditional individual gene over-representation based method using the standard hypergeometric test. In gProfileR, *p*-values are corrected by default using a unique multiple testing correction method (MTC), called the Set Counts and Sizes (SCS) procedure, which is analytically derived from extensive simulation experiments and purports to account for “the actual structure behind functional annotations”. In other words, issues relating to the overlapping structures within annotation repositories are believed to be addressed indirectly and implicitly, as a special case of MTC. We use gProfileR in the simulation experiment. For completeness, we also list gProfileR’s results on the three biological datasets evaluated here; however, some of the pathways listed by gProfileR are very recent additions to the KEGG repository that are as yet not available in other tools, including our current implementation of SIGORA.

#### DAVID (http://david.abcc.ncifcrf.gov/(Huang, Sherman & Lempicki, 2009; Jiao et al., 2012))

DAVID provides individual-gene pathway analysis over-representation analysis using the EASE Score, a modified Fisher Exact *p*-value that is designed to raise the bar for statistical significance of smaller pathways. This avoids situations in which observation of only a few genes from a small pathway would make it equally (or even more) significant than observing dozens of genes from a larger pathways. The EASE Score is generally more conservative than the Fisher Exact *p*-values.

Apart from this modification, DAVID’s *functional annotation charts* implement a traditional individual gene over-representation based method that treats all genes equally. DAVID has also introduced the concept of *functional annotations clusters* that are motivated by the idea that rather than focusing on significance of individual pathways, the true nature of a phenotype should be examined by considering the overall emerging picture of interrelated pathways. In some situations, clusters of interrelated pathways can be considered collectively significant while some (or most) of the individual pathways in those clusters might fall slightly beyond the significance threshold. Although this is undoubtedly a sensible statement, the measure of *interrelatedness* used in DAVID’s functional clusters is in diametrical contrast to the reasoning behind SIGORA: DAVID’s authors postulate that similar pathways tend to contain similar gene members. In DAVID’s functional annotation charts, the more common genes annotations share, the higher chance they will be grouped together as interrelated pathways, and the better the chances of the emerging cluster to become (collectively) significant.

For the simulation experiment, we will focus on DAVID’s functional charts. In our evaluation of analysis results on biological datasets, we will also briefly exemplify the pitfalls of DAVID’s Functional clusters.

#### GSEA (Subramanian et al., 2005)

The three methods discussed above (InnateDB, gProfileR and DAVID) are over-representation based methods that (like SIGORA) operate on a pre-filtered list of genes of interest (query list). The list of genes of interest is often determined using a (combination of) threshold(s) (e.g., fold change and *p*-value of differential expression). The *p*-values are in essence derived from a contingency table.

GSEA, in contrast, is the most prominent representative of a very different category of pathway analysis tools that do not operate on a pre-selected list and do not use contingency tables. In GSEA, all genes in the dataset are first ranked by their difference regarding a single biological metric (e.g., signal to noise ratio) between the two conditions. This ranked gene list is then used to assign a normalized enrichment score (NES) – defined as the maximum deviation of a running sum statistic from zero, adjusted for the number of genes in the pathway – to each pathway. The statistical significance of the NES is determined by sample permutation (i.e., randomly exchanging the phenotype class labels).

We compare GSEA and SIGORA in the analysis of three biological datasets. Some of the observed differences in the results of GSEA analysis to SIGORA are inevitably due to the fundamental differences between ORA and GSEA methods. In particular, regardless of their biological relevance to the examined dataset (or lack thereof), the significance of some of pathways observed by GSEA is due to ‘subtle but coordinated changes’ in expression levels of genes that are not in the list of differentially expressed genes.

This issue equally applies to the two remaining methods, GSEA-PRERANKED and AF, which are described below.

#### GSEA-PRERANKED

As the name suggests, this is a variant of GSEA where the input format is not an expression matrix, but a pre-ranked list of genes. Accordingly, as there is no sample information available, the statistical significance is derived from gene set permutation instead of sample permutations. Technically, applying GSEA and GSEA-PRERANKED to the same dataset can lead to identical NES, but very different FDRs (http://www.broadinstitute.org/gsea/doc/GSEAUserGuideFrame.html). Hence, the recommended threshold for statistical significance is different (0.05 instead of 0.25). We compare GSEA-PRERANKED to SIGORA in analysis of simulation datasets. Like standard GSEA, GSEA-PRERANKED does not provide a mechanism for dealing with shared components of pathways.

#### Appearance Frequency modulated GSEA (*AF*) (Ma, Sartor & Jagadish, 2011)

*AF* is a recently proposed variant of GSEA that is explicitly designed to deal with issues posed by shared components. In this respect (the intended benefit), *AF* is the most similar method to SIGORA among all methods compared here. *AF* assigns weights to individual genes based on number of associated pathways and performs a GSEA analysis. Methodologically, *AF* inherits most of GSEA’s characteristics and is quite distinct from SIGORA. We compare *AF* and SIGORA in the analysis of three biological datasets.

### Simulation experiment

#### Creation of simulated input lists

As a preliminary measure to quantify the effect of shared components on the number of spurious pathways, we conducted a simulation experiment over 1,000s of simulated gene lists that are created by applying the following procedure: From a set of 175 human KEGG human pathways that are in the repository of all four compared tools (SIGORA, DAVID, gProfiler and GSEA_preranked), *n* pathways are chosen at random and a fraction (*alpha*) of genes in each selected pathway are marked as differentially expressed (DE). The restriction to 175 common pathways is intended to reduce the effects of diverging update-status across analysis tools. The list of DE genes from five selected pathways is used as a query list for SIGORA, gProfiler and DAVID. To create an input list for GSEA_preranked, a score of 2 is assigned to the selected DE genes and a score of 1 to all remaining human genes. This procedure is repeated 1,000 times at fixed values for *alpha* and *n*.

### Biological datasets

We further compare each of the different pathway analysis tools by examining their results when applied to three different gene expression datasets. These datasets incorporate the results of microarray studies investigating the host response to a parasite infection (Experimental Cerebral Malaria), a viral infection (Dengue Fever), and a bacterial infection (Tuberculosis) (Lovegrove et al., 2007; Thuong et al., 2008; Hoang et al., 2010). We map the lists of differentially expressed genes in each experiment (as provided by the authors of the respective studies) to unique Ensembl/Entrez IDs and use the resulting sets as input lists for four over-representation based methods (InnateDB, DAVID, gProfiler and SIGORA), ensuring that all methods are run on identical input lists. Additionally, two GSEA-based methods (GSEA and AF) are also applied to the corresponding expression datasets obtained from the gene expression omnibus (GEO): GSE25001, GSE11199, GSE11199.

## Results

### Results on simulated gene lists

To evaluate the performance of SIGORA, we compared it to three other popular pathway analysis methods (DAVID, gProfiler and GSEA_Preranked) applied to simulated data, where we know *a priori* which are the significant pathways. The simulated input data was created by randomly choosing five KEGG pathways and selecting a fraction (alpha, 50% or 15%) of the genes in each of these pathways as being “differentially expressed”. Each of the methods was then applied to the selected gene list to determine the statistically significant pathways (using the respective recommended significance threshold and MTC approach, see Text S1).

If the sharing of genes between different pathways was not a factor, we would expect that each method should identify only the five preselected pathways as significant. As can be seen in Table 3, in our experiments (using 1,000 simulated datasets with alpha = 50% and 1,000 datasets with alpha = 15%), this was not the case: gProfileR, for example, identified more than 60 pathways on average as being significant at alpha = 50%, despite only 5 pathways being simulated as significant in the input data. SIGORA performed best by this measure and identified on average 8 pathways as significant across the different datasets.

**Table 3:** Performance metrics for several pathway analysis methods run on 1,000 simulated gene lists at two different alphas (15% or 50%). Each of the simulated datasets contained either 15 or 50% of genes in 5 randomly chosen pathways. For each analysis method and each input gene list, the identified (statistically significant) pathways were recorded and the Precision *(true positive results/all significant pathways)*, Recall *(true positive/chosen)* and F1 score (*harmonic mean of Precision and Recall*) were calculated by comparing the list of the (*n*) chosen pathways with the list of identified pathways. More specifically, for the purpose of this analysis, a statistically significant pathway is considered a *true positive* if it is among the five chosen pathways and a *false positive* otherwise; furthermore, any chosen pathways that are not identified by a method as statistically significant are considered *false negatives*. The entries in bold show the method with the best performance according to each measure.

Alpha	Method	Average number of significant pathways	Average Recall	Average Precision	F1 score (harmonic mean of Precision and Recall)	Average rank of the original pathways within the analysis results
15%	DAVID	11.22	0.71	0.32	0.44	6.8
	gProfiler	34.17	**0.95**	0.14	0.24	7.8
	GSEA_Preranked	0.09	0.01	**0.74**	0.03	29.6
	SIGORA	**6.41**	0.72	0.56	**0.63**	**3.6**
50%	DAVID	30.25	0.98	0.16	0.28	6.8
	gProfiler	62.48	**0.99**	0.08	0.15	8
	GSEA_Preranked	13.91	0.87	0.32	0.46	5.6
	SIGORA	**8.87**	0.89	**0.50**	**0.64**	**3.7**

The Recall and Precision metrics in the third and fourth columns of Table 3 capture the relationship between originally preselected ‘target’ pathways and the identified pathways. More specifically: Recall describes the fraction of the target pathways that were identified as significant and Precision signifies the fraction of statistically significant pathways that were among the originally selected pathways. Neither of these two metrics by itself is decisive, and there is a certain trade-off between the two metrics. This trade-off is captured by the F1-score, the harmonic mean of Precision and Recall. As can be seen in Table 3, SIGORA had the best F1 scores when compared to the other methods.

Finally, note that although this analysis is based on recommended significance thresholds in each method, Precision, Recall and F1 score are all dependent on the – ultimately arbitrary – choices of significance thresholds. The last column in Table 3 describes the results according to a less threshold-dependent measure. Ideally, the preselected pathways would occupy the first five positions in the list of the identified pathways, resulting in an average rank of 3. As can be seen in the last column of Table 4, at both choices for alpha (15% or 50% of the genes in each pathway selected) the average rank of the originally preselected pathways (‘target pathways’) in SIGORA’s results (3.6 and 3.8 respectively) is very close to this ideal value, whereas the other methods tend to identify several additional pathways as more significant than the target pathways, resulting in higher average ranks of target pathways in these methods.

**Table 4:** List of all pathways identified as statistically significant by each method compared in this study and their respective ranks (by *p*-value) in the analysis of a Dengue fever gene expression dataset. The entries in bold are significant largely due to sharing genes with other more relevant pathways.

	DAVID	GSEA	AF	gProfileR	InnateDB	SIGORA
**Systemic lupus erythematosus**	**1**	**3**	**2**		**1**	**1**
Hepatitis C					10	2
Complement and coagulation cascades	2			3	3	3
RIG-I-like receptor signaling pathway		1	3		9	4
Cytosolic DNA-sensing pathway		2	1		4	5
Osteoclast differentiation					7	6
Chemokine signaling pathway			6			7
Lysosome						8
Antigen processing and presentation			9			9
NOD-like receptor signaling pathway		4	4			10
Toll-like receptor signaling pathway		6	8		5	11
**Staphylococcus aureus infection**					**2**	
Long term potentiation			10			
**Leishmania infection**		**5**	**11**			
Ribosome			5			
**Malaria**					6	
Allograft rejection			7			
**Prion diseases**			**12**		**8**	
Measles				1		
Influenza A				2		
Herpes simplex infection				4		
**Pertussis**				**5**		

### Results on published datasets

In addition to the simulated data, we also compared SIGORA to five different methods (InnateDB, DAVID, gProfileR, GSEA and AF) applied to real biological data, in this case three different gene expression datasets. Full details of the input genes, *p*-values and highlighted pathways obtained by each method can be found in the Tables S1–S3.

#### Tuberculosis

**Figure 4:**
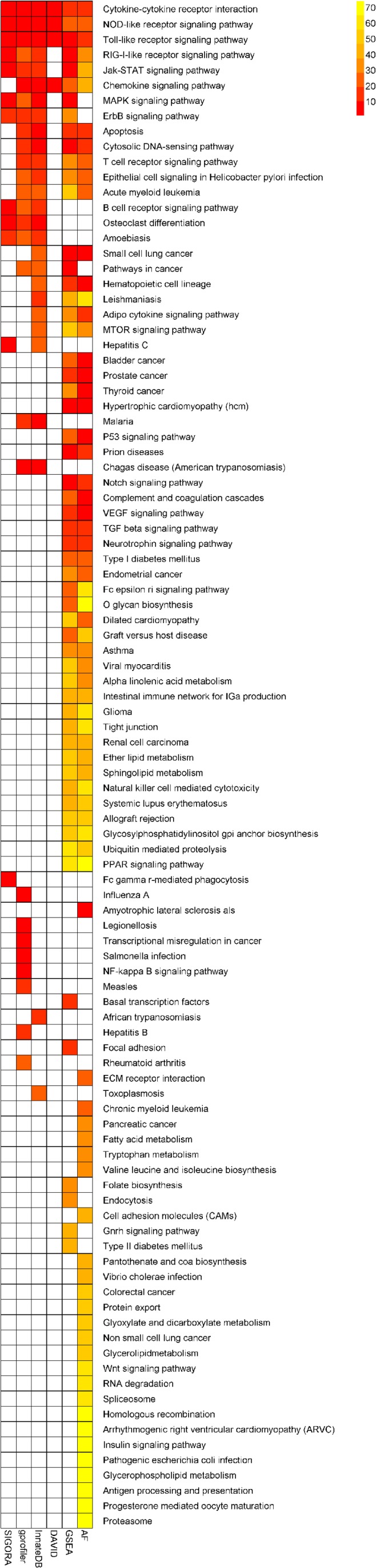
Results of six different pathway analysis methods applied to a gene expression dataset measuring the host transcriptional response to *M. tuberculosis* infection of human macrophages. The heatmap shows all pathways that were identified as statistically significant by at least one of the six different pathway analysis methods. The more red the color the higher the rank of that pathway for a particular method. The heatmap is sorted by the number of methods identifying a particular pathway as significant.

**Figure 5:**
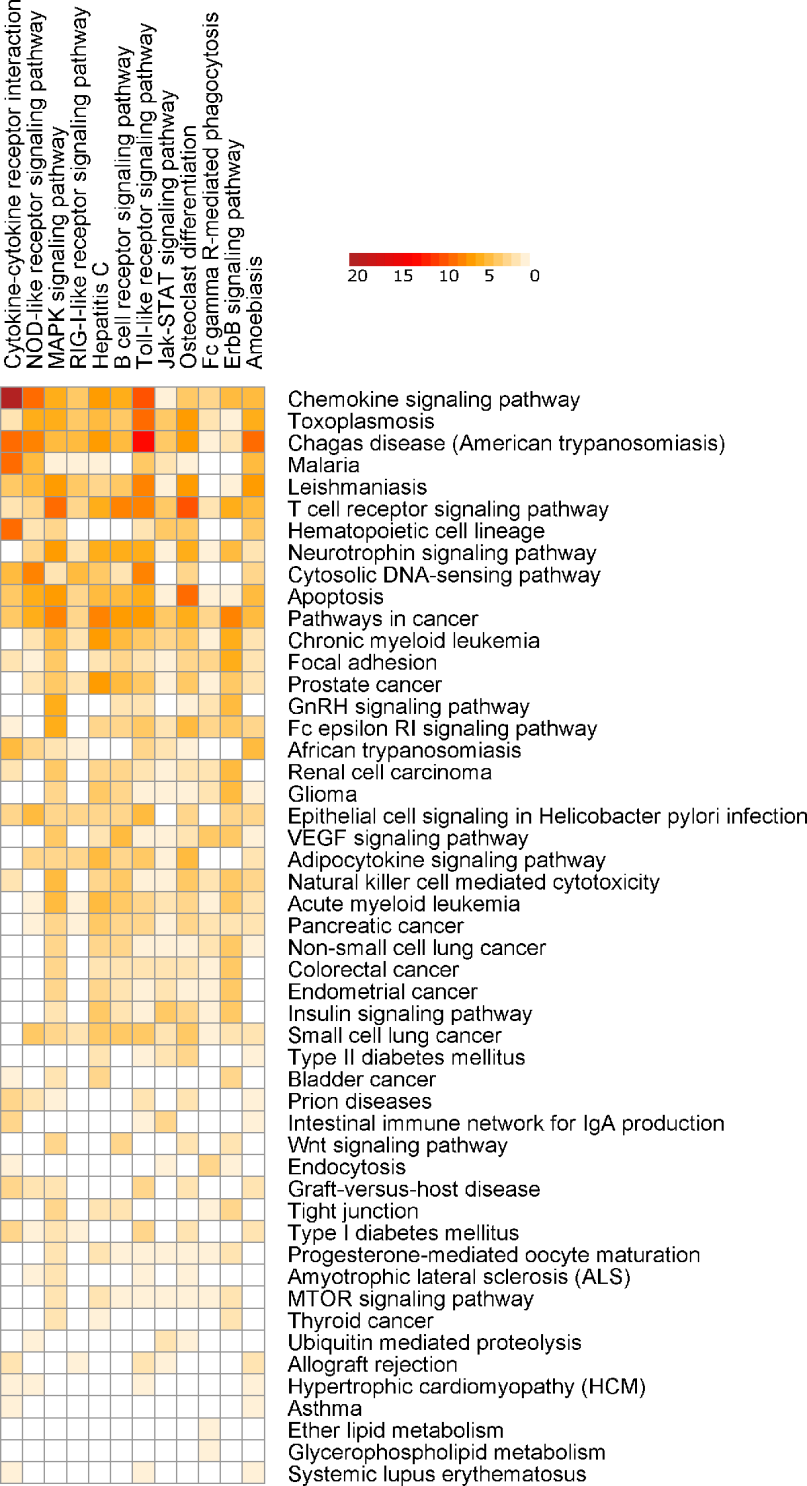
Number of differentially expressed genes that are shared between SIGORA’s pathways (vertical axis, ordered by rank) and additional pathways identified as significant by other methods on the TB dataset.

SIGORA was compared to five different pathway analysis methods (InnateDB, DAVID, gProfileR, GSEA and AF) applied to a gene expression dataset (GSE11199) which measured the host transcriptional response in human macrophages infected with *Mycobacterium tuberculosis* (Thuong et al., 2008). 1,250 transcripts (corresponding to 1,100 distinct Ensembl genes) were identified by Thuong et al. as being induced in response to this infection. Figure 4 shows the pathways that were identified as statistically significant by each of the six methods. The first thing that one notes is that the GSEA-based methods tended to predict large numbers of pathways as statistically significant (the AF method predicted nearly a third of the KEGG database as significant in this example). This is somewhat by design, as GSEA methods attempt to identify subtle but coordinated changes in gene expression. This may be very helpful in investigating cases where there are only subtle differences between conditions but in a dataset like this one, it leaves the biologist bewildered as to which pathways should be followed-up on experimentally. In the other extreme is DAVID, which predicted only 4 pathways as statistically significant. SIGORA, on the other hand, identified 12 pathways as statistically significant; 10 of which were also identified by at least two other methods. Comparing the pathways identified by SIGORA as significant to the significant pathways identified by the other methods, one can see (Fig. 5) that many of the pathways identified by other methods as significant but not by SIGORA share many genes with the SIGORA pathways. Notably, after removing the multifunctional genes that are involved in the pathways identified by SIGORA from the input list, the individual gene over-representation based methods (DAVID, InnateDB and gProfileR) did not return any significant pathways at all. This reinforces our observation from the simulated data that SIGORA will identify truly significant pathways but avoid identifying pathways that are significant because they share genes with other more relevant pathways.

Interestingly, SIGORA may also be able to identify some important pathways that are not significant using other methods. One pathway was identified as significant in this dataset only by SIGORA; Fcγ*R*-*mediated phagocytosis*. Fcγ receptors regulate immune activation and susceptibility during *Mycobacterium tuberculosis* infection (Maglione et al., 2008; Maertzdorf et al., 2011) and it has been implied that “entry through Fcγ receptors may specify a distinct intracellular trafficking pathway for virulent *M. tuberculosis”* (Ernst, 1998). Finally, DAVID’s top *functional cluster* for this dataset (Enrichment score 0.99) contained 16 pathways, 12 of which are different cancer subtypes (Table S1).

Table S1 lists all Pathways identified as statistically significant by each of the considered methods and their respective ranks (by *p*-value) in analysis of this dataset.

#### Experimental cerebral malaria

Example 2 is a mouse cerebral malaria (ECM) dataset, comparing the whole-brain transcriptional responses of genetically susceptible (C57BL/6) and resistant (BALB/c) inbred mouse strains 6 days after infection with Plasmodium *berghei* ANKA (NCBI GEO: GSE7814) (Lovegrove et al., 2007). We fist performed a differential expression analysis using Geo2R to obtain a list of up-regulated genes at FDR <0.01 (637 Ensembl genes, Table S2). We use this list as input for SIGORA, InnateDB, gProfileR and DAVID, and apply GSEA and AF to the corresponding expression matrix.

**Figure 6:**
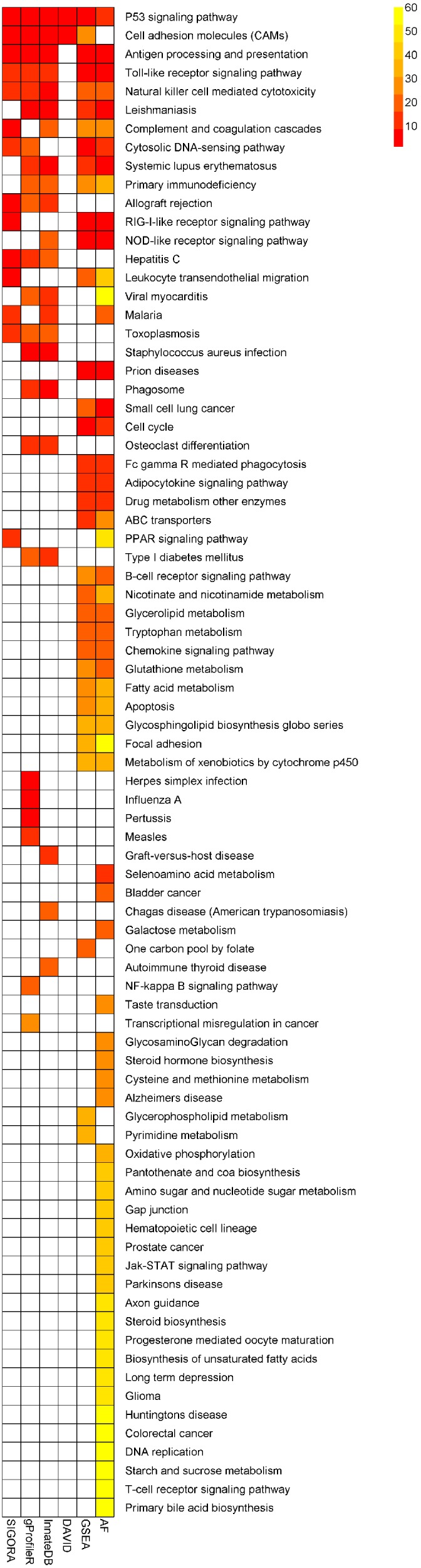
Comparison of results of six different methods on a mouse experimental cerebral malaria dataset. The heatmap shows all pathways that were identified as statistically significant in at least one of five different pathway analysis methods. The more red the color the higher the rank of that pathway for a particular method. The heatmap is sorted by the number of methods identifying a particular pathway as significant.

Similar to the previous example, the number of KEGG pathways that different methods identified as statistically significantly enriched in this dataset varied widely: AF identified 59 pathways (out of 185 in its repository), while DAVID highlighted just two pathways. SIGORA identified 14 pathways as significant, 13 of which were also reported by at least two other methods (Fig. 6, Table S2). The remaining pathway is *PPAR signaling pathway* (discussed further below).

Aside from this big-picture view, the strong changes in the rank orders of the following individual pathways are also worth mentioning:

#### Complement and coagulation cascades

This pathway is the 6th ranked pathway in SIGORA’s results. Three additional tools (InnateDB, GSEA and AF) also identify this pathway as significant, but only at considerably lower ranks (the 17th, 25th and 27th position, respectively). Complement and coagulation pathways have been shown to be critically involved in the development of ECM (Van der Heyde et al., 2006; Francischetti, Seydel & Monteiro, 2008; Ramos et al., 2012).

#### Leukocyte transendothelial migration

This pathway is the 7th ranked pathway in SIGORA’s results, the 23rd ranked pathway in GSEA and the 44th ranked pathway in AF. The remaining methods do not identify this pathway as statistically significant. Polymorphonuclear leukocyte recruitment has been shown to be responsible for increased permeability of the blood–brain-barrier, and is strongly associated with fatality rates in ECM (Senaldi et al., 1994; Bell, Taub & Perry, 1996).

#### PPAR signaling pathway

This pathway is at position 14 of SIGORA’s results. The only other method to identify PPAR signaling is *AF*, at position 47. Targeting of PPAR is currently being explored as a novel adjunctive therapy for cerebral malaria (Balachandar & Katyal, 2011; Serghides, 2012). Notably, PPARγ has been reported to be one of only two genes in a cerebral malaria-resistance locus identified using a genome-wide analysis of 32 different inbred mouse lines (Bopp et al., 2010) and modulation of the inflammatory response to *P. berghi* infection by an antagonist of this gene has greatly enhanced the survival rates in mice (Serghides et al., 2009).

At the same time, SIGORA avoids a few biologically implausible pathways that are considered highly significant by at least two other methods: e.g., *“Staphylococcus aureus infection”* (a bacterial infection) is the most significant pathway in InnateDB’s results, and the second highest ranked pathway in gProfileR’s results, but is not significant in SIGORA’s results. Similarly, GSEA and AF both identify *“Prion diseases”* as significant (position 2 and 4, respectively) and again, this pathway is not significant in SIGORA’s results. Other examples include: *“Small cell lung cancer”* (GSEA, AF), *“Viral myocarditis”* (InnateDB, gProfileR, AF), *“Type I diabetes mellitus”* (InnateDB, gProfileR) (Fig. 6 and Table S2).

DAVID’s top ranking functional cluster for this dataset (Enrichment Score: 2.7) groups *“Antigen processing and presentation”*, “*Viral myocarditis”, “Allograft rejection”, “Graft-versus-host disease”, “Type I diabetes mellitus”* and *“Autoimmune thyroid disease”* together (Table S2). All of these pathways having highly overlapping annotations.

#### Dengue fever

As a third evaluation set, we re-examined a list of 483 up-regulated genes in the whole blood transcriptome of patients infected with dengue virus (NCBI GEO: GSE25001) (Hoang et al., 2010). More specifically, we compared expression profiles of hospitalized patients with uncomplicated Dengue during acute phase (≤72 h of illness history) to follow up samples of such subjects two weeks after discharge (*n* = 72).

**Figure 7:**
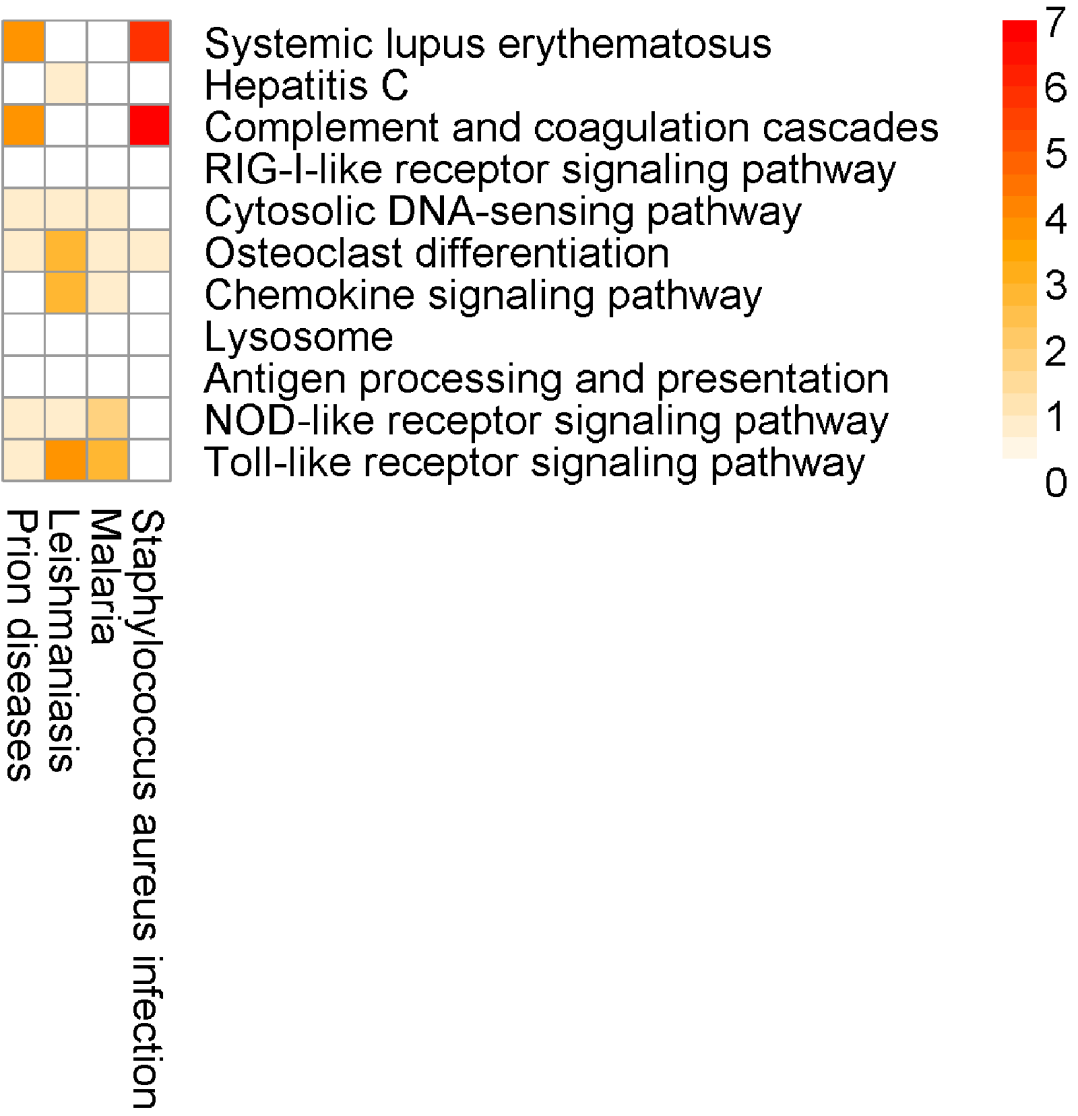
Number of differentially expressed genes that are shared between SIGORA’s pathways (rows, ordered by rank) and additional pathways by other methods (columns) in the analysis of the Dengue dataset.

Here, for the most part, the SIGORA results contain well-defined immunity related pathways. As before, some additional, potentially spurious pathways that are identified by other methods are not significant in the SIGORA analysis. Examples include “*Staphylococcus aureus infection*” (the second ranked pathway in InnateDB results) and the “*Prion Disease*” pathway (identified by both InnateDB and AF). These two pathways share components with the complement pathway (4 and 6 up-regulated genes respectively, Fig. 7), which has been shown to have a role in neutralising Dengue (Shresta, 2012).

Again, this example contains a possibly relevant pathway that was significant in SIGORA’s results, but overlooked by the other methods: *“Lysosome*”, at position eight of SIGORA’s results (Table 4, Table S3). Recent experimental evidence suggests that manipulation of the host’s autophagolysosomes by the dengue virus is an important part of the virus’s life cycle (Khakpoor et al., 2009; Heaton & Randall, 2010).

DAVID does not return any functional clusters for this dataset.

### Discussion and related work

The existence of shared components between pathways poses a challenge for pathway analysis methods: which of the statistically significant pathways associated with such components are the most biologically significant? In 2005, Khatri and Drăghici surveyed the state of the art analysis methods and tools of the time and outlined several limitations as the challenges for the next generation of analysis tools. One such challenge “*is related to genes that are involved in several biological processes. For such genes, all current tools weight all the biological processes equally. At the moment, it is not possible to single out the more relevant one by using the context of other genes [of interest] in the current experiment*” (Khatri & Drăghici, 2005).

The years since have seen many interesting developments in the functional analysis of high throughput biological data: Several methods (Alexa, Rahnenführer & Lengauer, 2006; Grossmann et al., 2007; Jupiter, Sahutoglu & VanBuren, 2009) have been described to highlight processes at the appropriate level of specificity and to reduce the redundancy of the results in the context of Gene Ontology (GO) analysis, where the overlap between categories are often due to the hierarchical organization of the ontology. These methods, however, fail to account for overlap between pathways that don’t involve the full inclusion of all members of a pathway in another pathway. To deal with such cases, Antonov et al. (2008) proposed the creation of new functional categories as complex Boolean combinations of available GO terms. Unfortunately, such combinations are often not easy to interpret and a comprehensive search over all possible combinations is computationally infeasible.

Outside of GO, a few methods have been proposed that indirectly tackle the issue by either discriminative treatment of individual genes or alternative representation of the pathway repository in specific scenarios. An example of the later approach (alternative representation of the pathway repository) is Bayesian Pathway Analysis, BPA (Isci et al., 2011). BPA transforms each pathway in a pathway repository into a separate Bayesian Network (BN) and scores the fit of each model with the experimental (expression) data. BPA is expected to leverage the expression status of other genes in the experimental context, as BNs, in contrast to simple lists of genes, are deemed capable of accommodating local interactions between genes. Although highly sophisticated, BPA is computationally intensive and by design limited to the interpretation of expression datasets.

An early example of the former approach (non-egalitarian treatment of individual genes) is impact-analysis (Draghici et al., 2007). Impact analysis integrates the magnitude of each gene’s expression change along with the type (e.g., receptor, transcription factor) and position of each gene within the given pathways and their interactions into the statistical framework, however, the authors do not explicitly address the problems related to component sharing among pathways.

More recently, it has been proposed to add an appearance frequency based parameter to the statistical framework of GSEA. This additional parameter is intended to weaken the contribution of genes with multiple pathway memberships to the statistical significance of all of their associated pathways (Ma, Sartor & Jagadish, 2011). While this is a significant step in the right direction, the addition of such a parameter does not exploit the status of other genes in the experiment for the selection of the most relevant function of a gene in the experimental context. As exemplified by the gene, *BRCA1* in Khatri & Drăghici (2005), even key players of one process (maintaining genomic stability) can have several less prominent roles in other unrelated pathways (response to nutrient and brain development). Nor can appearance frequency distinguish between the causes of appearance of a gene in several pathways, which, aside from functional pleiotropy of genes, can be partly due to the hierarchical organization of some pathway repositories like REACTOME and GO.

Here we introduced the concept of Pathway-GPS, as genes or gene-pairs that (as a combination) are specific to a single pathway, and we described Signature Over-representation Analysis (SIGORA) as a novel (and comparably efficient) approach to pathway analysis. SIGORA uses Pathway-GPS to bridge the gap between the context sensitive and collaborative nature of biological processes on one side and the universal and discrete statistical framework of over-representation analysis on the other side. Although each Signature’s weight is fixed in advance, the net contribution of an individual gene *G* to the measured success (parameter *k* in Table 1) of each of its associated pathways is not fixed and depends explicitly on the status of its partners (genes that together with *G* form a GPS).

In contrast to the GSEA-based solutions, SIGORA inherits the versatility of the ORA statistical framework and is applicable to lists of genes of interest obtained in any type of high throughput experimental set-up (e.g., copy number variations from cellular profiling, lists of epigenetically silenced genes from promoter methylation analysis, differential gene expression data from NGS and microarrays or SNPs from GWAS experiments) without the need for adaptation of the computational method. This is especially notable in situations where ranking of the entire dataset by a single biological parameter (as required by GSEA) is not feasible (see Huang, Sherman & Lempicki, 2009, for a few examples).

## Conclusions

This paper highlights the level of component sharing between pathways and demonstrates how this can lead to misleading/spurious results in current pathway analysis approaches that treat all pathway members as equally informative. Here we introduce a novel approach that accounts for the overlapping structure of pathway annotation by focusing on unique features (‘*Signatures*’) of pathways. To our knowledge, this is the first over-representation based method to do so.

Applied to several published datasets, our approach highlights biologically meaningful processes that would otherwise fall below statistical significance thresholds, and avoids some of the biologically implausible processes highlighted by other methods. This suggests that our approach delivers a useful complementary tool for pathway analysis.

## Supplemental Information

10.7717/peerj.229/supp-1 Text S1 This file gives a short overview of respective limitations of ORA and GSEA based pathway analysis frameworks, discusses the need for using gene-pair combinations (as opposed to PUGs only) and the viability of higher order combinations (three or more genes)

10.7717/peerj.229/supp-2 Figure S1 Signature Transformation of a hierarchically organized pathway repository as an iterative process. Here, G0 to G7 are genes that are annotated in the hierarchically organized pathways P1 to P5, as shown in the Vann-Diagram (inset). In the first iteration, only signatures for the outer-most level of the hierarchy(P5) are determined. The GPS for the higher levels are the ones associated with the less general pathways, and are only visible after removal of the more general terms. For example, the GPS of P1, P2 and P3 are only visible at level 3, after removal of P5 and P4.

10.7717/peerj.229/supp-3 Table S1 Detailed results for the pathway analysis of a TB expression dataset (GSE11199) by 6 different methods. The worksheet contains 9 tabs: the list of the input genes, the full results of each of the six compared analysis tools for this data-set, a summarizing 6way-comparison sheet of the ranks of pathways that were identified as significant by at least one method, and an additional tab for DAVID’s functional clusters.

10.7717/peerj.229/supp-4 Table S2 Detailed results for the pathway analysis of a mouse experimental cerebral malaria (ECM) expression dataset (GSE7814) by 6 different methods. The worksheet contains 9 tabs: the list of the input genes (for the ORA-based methods), the full results of each of the six compared analysis tools for this data-set, a summarizing 6way-comparison sheet of the ranks of pathways that were identified as significant by at least one method, and an additional tab for DAVID’s functional clusters.

10.7717/peerj.229/supp-5 Table S3 Detailed results for the pathway analysis of a Dengue fever dataset (GSE25001) by 6 different methods. The worksheet contains 8 tabs: the list of the input genes, the full results of each of the six compared analysis tools for this data-set, a summarizing 6 way-comparison sheet of the ranks of pathways that were identified as significant by at least one method.
