# Supplementary material for: Pathway-GPS and SIGORA: identifying relevant pathways based on the over-representation of their gene-pair signatures
Source: PeerJ. 2013 Dec 19;1:e229. doi: 10.7717/peerj.229 (PMC3883547; doi:10.7717/peerj.229)
Supplement: Text S1 [file peerj-01-229-s001.docx]

**Supplementary Text for "Pathway-GPS and SIGORA: Identifying relevant pathways based on the over-representation of their gene-pair signatures"**

**Limitations of ORA and GSEA frameworks**

Pathway ORA methods investigate whether the observed fraction of genes belonging to a specific pathway in a user specified list is more than one would expect by chance. The genes in the input list are usually determined based on an arbitrary threshold for significance, for example, genes that are significantly differentially expressed or genes that are significantly associated in a GWAS. GSEA methods, on the other hand, do not apply such a threshold and instead consider the collective rank of all genes in a given set (e.g. a pathway). GSEA purports to avoid some type II (false negatives) errors of ORA approaches by accounting for subtle but coordinated differences in a given pathway between conditions of interest.

GSEA based methods are limited to experimental designs consisting of two groups of samples (e.g. infected vs. control). In many cases, the upstream data processing and comprehensive gene selection statistics cannot be simply avoided or replaced by GSEA, the effects of additional factors (e.g. gender, age) besides the assignment to one of the two groups cannot be taken into consideration, and the results of such preprocessing often don't conform to GSEA-required input data structures (Huang, Sherman, & Lempicki, 2009).

**Need for combinations**

A simplistic approach to the challenges posed by shared components would be to discard genes with multiple pathway annotations and to limit the analysis to the investigation of single characteristic genes that have only one pathway annotation (we call these *pathway unique genes*). This would, however, drastically reduce the discovery power of the analysis, because most genes are not pathway unique genes and many pathways do not have such markers. Furthermore, genes with multiple pathway annotations do contain valuable information on the underlying biological processes.

**Viability of higher order combinations as signatures (Sufficiency of Gene pairs)**

The move from *PUG*s to *GPS* is motivated by the observation that if the method were to focus exclusively on *PUG*s, pathway-membership information for a substantial fraction of human genes would be lost. Our analysis suggests that GPS deal with this issue rather effectively: for instance, all human genes with a KEGG pathway-annotation participate in at least 9 (in average, 382) KEGG-GPS, i.e. there are no ‘orphan’ genes.

A naturally arising question is: “*Would an extension of the signature concept to sets of more than two genes not provide even more information about the relevant pathways?”*

To answer this question, let us first recall that for a pathway containing *n* genes, there are (*n choose k*) distinct subset of size *k* and a total of 2^n^ -1 distinct non-empty subsets. (e.g. for a pathway with 102 genes –like TLR – there are around 5000 gene pairs , 171700 possible triplets, and over 4,2 million possible 4-tuples ). Overall, there are over 256 Million co-annotated triplets and approximately 62 Billion co-annotated quartets of human genes in KEGG.

The vast majority of these higher order combinations of co-annotated genes are bound to be specific to a single pathway, but this is a rather trivial effect: Expanding any GPS *g1,g2* by an additional gene *g3* from the same pathway would automatically create a new ‘triplet signature’ *g1,g2,g3* (otherwise *g1,g2* could not be a GPS). Only in an estimated 1.4 million out of 256 Million or 0.5% of all cases, these triplets would potentially contain novel information: (There are ~100,000 non-GPS co-annotated gene pairs, the average human KEGG pathway contains 16 genes, which means that each non-GPS pair can be expanded by -in average- 16-2=14 different genes to build a triplet).

In other words, the GPS capture at least 99.5% of the information that can be coded by triplet signatures, at a much smaller computational cost.

A more refined strategy would seek to identify triplets that genuinely carry novel information. In order for a set of more than two genes to provide new evidence (i.e. information that is not already captured by the GPS), such set would have to include three or more genes such that a) all the genes in the set co-occur in a single pathway *and* b) no combination of two of the genes in the set (e.g. triplet Signature) is a GPS. Although the second criterion reduces the number of candidate set, the resulting gene sets still do not add an actionable amount of additional information.

To further elucidate this point, we identified 619 ‘triplet signatures’ for the TLR pathway fulfilling the above criteria and involving AKT1. AKT1 is annotated in TLR and several other pathways. All TLR-GPS involving AKT1 contain a TLR-PUG, (i.e. no combination of AKT1 with another multi-pathway gene results in a TLR GPS). All 619 TLR-triplets contained AKT1 and two additional genes *g1, g2*, such that *g1,g2* was not a GPS of TLR (or in fact a GPS of any KEGG pathway). In total, 66 out of the 102 TLR genes were involved in these triplets. Using the list of these 66 genes (the building blocks of the signature triplets) as (the query list) input, SIGORA identified 169 TLR-GPS as present and declared TLR as significant. To understand this behaviour, consider two triples *g1,g2,g3* and *g1,g4,g5*. Although by construction we postulate that *g2g3*, *g1g3*, *g4g5* and *g1g5* should not be GPS, *g4g2*, *g5g2*, *g4g3* and *g5g3* still might be. For a concrete example, consider the triplet signatures (AKT1, **TRAF3,** **MAPK12**) and (AKT1, **CXCL11**, IL10RB) of TLR-signaling: although within each of these triplets, there is no gene-pair specific to any single pathway, (**MAPK12, CXCL11**) and (**TRAF3**, **CXCL11**) are TLR-GPS.

**Alternative weighting schemas**

In the following discussion, it is assumed that two genes, *g1 and g2* are annotated in *i and j* pathways, respectively, and that *g1* and *g2* only co-occur in a single pathway, *P*.

In the main text, we argue that: The GPS consisting of *g1* and *g2* should be assigned a weight (to quantify its reliability as an indicator of *P*), and that the weight of this GPS should monotonically decline with increasing *i* and *j.*

As it is often the case in harnessing information from projections of bipartite networks (Allali, Magnien, & Latapy, 2013; Padrón, Nogales, & Traveset, 2011) , there are many different plausible and ‘natural’ ways to quantify this intuitively clear notion that with increasing *i* and *j*, the reliability of (*g1,g2*) as an indicator of *P* monotonically decreases.

We have explored five different such weighting functions (**Figure below**).

**
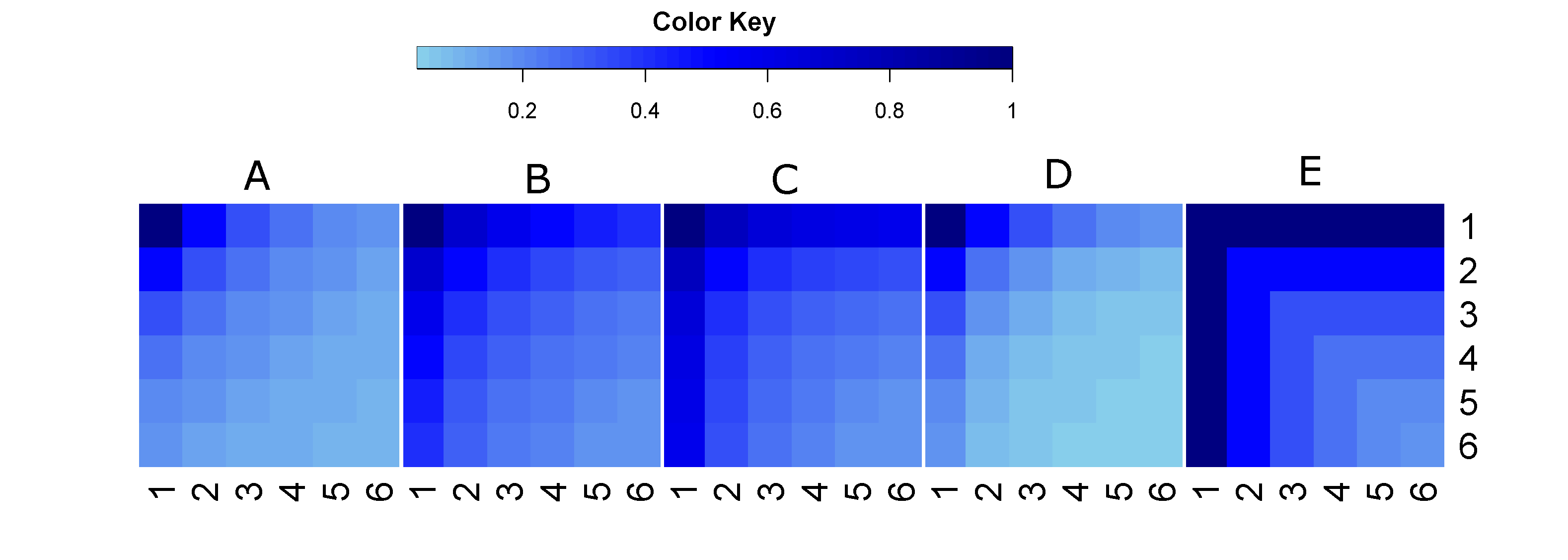
Embedded Figure:** The monotonic decline of five alternative GPS-weighting schemes with increasing *i* and *j*. Only the values for *i* and *j* up to six are illustrated.

A: Jaccard B: cosine C: inverse harmonic mean D: independent decisions E: topological overlap.

1. $\frac{1}{i+j-1}$: The Jaccard similarity of *g1* and *g2.* (Number of common annotations of *g1* and *g2* , which is by definition of *GPS* always 1, divided by the total number of annotations of *g1* and *g2*.) The inverse of total number of pathways annotations of *g1* and *g2* (considered *individually*).
2. $\frac{1}{\sqrt{i*j}}$ : The cosine normalization of *i* and *j.*
3. $\frac{1}{2}*\left(\frac{1}{i}+\frac{1}{j}\right)$: The inverse of the harmonic mean of *i* and *j.*
4. $\frac{1}{i*j}$ : The reciprocal of the product of *i* and *j.*
5. $\frac{1}{\min(i,j)}$: The topological overlap of *g1* and *g2*: number of common pathways (by definition of *GPS* always 1) divided by *min (i,j).*

All of these functions are plausible weighting schemes and each has its own strengths and limitations. For example, the weighting scheme D corresponds to the probability that, assuming that *g1* and *g2* *uniformly and independently* ‘decide’ to engage in one of their annotated pathways, they both choose *P*. At the same time, the independence assumption in this scheme seems biologically unsupportable.

In practical terms, the functions (A and D) do not seem very useful as they decline rapidly with increasing *i* and *j* (**Embedded Figure**). In these schemes, for most possible values of *i* and *j*, the resulting weights are very close to zero and hardly distinguishable from each other. There are also additional epistemic and biological reasons in favor of a less stringent penalty for GPS that involve genes with higher *i* and *j*.

Similarly, weighting scheme E could be interpreted as the possibility that any of the two constituent genes ‘regulates’ *P*. In this scheme, however, all GPS with the same value of *min(i,j)* obtain the same weight, regardless of their respective values for *max(i,j),* which again does not necessarily result in extracting most information out of the query genes in a mathematically sound manner (**Embedded Figure**).

Among the functions listed above, the weighting function C seems to offer a more gradual and fine grained monotonic decline (**Embedded Figure**). It corresponds to a normalized voting scheme in (Allali et al., 2013) or to the shared visits model in (Padrón et al., 2011). Figuratively, one could think of *g1* and *g2* (the two genes in the GPS) as actors collaborating towards a common goal (the common pathway *P*): *g1* commits 1/*i* of its resources to *P*, while *g2* assigns 1/*j* of its resources to *P*. The function under (C) is the average commitment of *g1, g2* to *P.*

In addition to the above practical reasons against some alternative (albeit plausible) weighting schemes (that would result in a steeper decline of the weights), there are two additional reasons in favor of the more gradual decline (as exhibited by the inverse harmonic mean model that is used by default in SIGORA):

1) The “*correlated expression problem*”: Goeman and Bühlmann (Goeman and Bühlmann, *Bioinformatics,* 2007) have argued that the statistical framework of Over-representation based methods is flawed: In their view, Over-representation analysis does not account for the fact that changes in the expression levels of a gene are not random and independent of expression levels of other genes, as genes are often subject to common regulatory mechanisms. While this critique seems particularly convincing in the case of the individual gene ORA, one can argue that if *g1* is member of *i* pathways and *g2* member of *j* pathways, and *g1* and *g2* have a single common pathway, then there are *i+j*-1 counter-examples to the assumption that *g1* and *g2* are subject to exactly the same transcriptional regulatory mechanisms. Notably, this type of “evidence for transcriptional independence of *g1* and *g2” strengthens with increasing i and j.*

2) The “*knowledge bias problem*” Some well studied genes might be annotated in a relatively large number of pathways, in part because these genes have been known for a longer time and been subjected to more intensive scrutiny, and conversely, other genes might be annotated in only a few pathways, simply because they have not been a research focus.

Paradoxically, these considerations suggest that a GPS with rather large *i* and *j* could be a relatively *reliable* indicator of *P,* because the co-annotation of *g1* and *g2* in *P* (and only *P*) is less likely to be due to gaps in the state of our knowledge about pathway annotation of *g1* and *g2*, and less likely to be due to the transcriptional co-regulation of *g1* and *g2*.

Hence, a gradually declining weighting scheme (as used in SIGORA) seems overall more appropriate than the more steeply declining alternatives. Nevertheless, in our implementation, the user also has the option to use any of the other weighting schemes mentioned above or assign a constant weight of 1 to all GPS (This is implemented in *sigs_other_weights* function). Certain user-defined weighting schemes are also supported.

**Coexpression and co-annotation**

As mentioned in the previous section, correlated expression of genes has been long considered a methodological challenge to ORA-based methods, where the statistical framework presupposes sampling from an independent and identically distributed gene pool (Goeman & Bühlmann, 2007).

To determine whether Pathway-GPSs were more or less likely to be compounded by co-expression biases than traditional methods, we investigated the relationship between the number of shared pathway annotations and correlated gene expression on a repository wide scale (in KEGG) , using the ‘human top three highest correlated genes’ list from COXPRESdb (Obayashi & Kinoshita, 2011). COXPRESdb annotates the top three most correlated genes for each of over 19,000 genes, across hundreds of samples. The results can be summarized as follows: as is to be expected, highly co-expressed gene pairs tend to share pathway affiliations at much higher rates than randomly selected pairs of gene. However, if we limit the analysis to gene-pairs that have at least one pathway annotation in common, then highly co-expressed gene-pairs are more likely to be found among gene-pairs that share multiple annotations than those having just one shared annotation. In other words, Pathway-GPSs are less likely to display highly correlated expression behavior.

We were able to replicate these results in a case by case examination of several published lists of differentially expressed (DE) genes for individual pathological conditions. We observed that although such lists often contained many (hundreds) gene pairs from the above COXPRESdb-list, only a fraction (i.e. less than 10%) of such pairs were comprised of genes that shared a single pathway annotation. For example, DE genes in GSE781 (the dengue fever data set) contained 304 correlated gene pairs, only 22 of which were ‘Signatures’. In the implementation, the user can examine the proportion of highly correlated genes and gene-pair signatures in a dataset (by executing *coexpress-sigs* after *sigs*).

**Overview of pathway analysis methods compared in this study:**

In the main manuscript, we compare SIGORA to several other analysis methods. In those comparisons, the choices for the significance threshold and the multiple testing correction approach for each method are based on the recommendations of the authors of the respective method. The following table summarizes these choices.

Overview of pathway analysis methods that are compared to SIGORA in this paper.

| *Method* | *Reference* | *MTC and Threshold* |
| --- | --- | --- |
| *DAVID v.6.7* | (Huang et al., 2009; Jiao et al., 2012) | *FDR < 0.05* |
| *gProfiler* | (Reimand, Kull, Peterson, Hansen, & Vilo, 2007) | *g:SCS < 0.05* |
| *GSEA_Preranked* | (Subramanian et al., 2005) | *FDR< 0.05* |
| *GSEA* | (Subramanian et al., 2005) | *FDR <0.25* |
| *GSEA_AF (AF)* | (Ma, Sartor, & Jagadish, 2011) | *FDR <0.25* |
| *InnateDB* | (Lynn et al., 2008) | *FDR < 0.05* |

**Alternative evaluation criteria**

*Reproducibility of results across independent datasets* One criterion for measuring the reliability of a method is the consistency of its results for two (or more) biologically similar yet independently created datasets. In such a setup, the method is applied to both datasets independently and the number of (relevant) significant pathways that are identified in both datasets is determined. The idea is that the higher the number of such common pathways, the more robust the method. Ma et al used this criterion to compare GSEA and AF (Ma et al., 2011). More specifically, they applied both GSEA and AF to histological grade 1 *vs.* grade 3 ER+ tumors from GSE3494 (Miller et al., 2005) and histological grade 1 *vs.* grade 3 ER+ tumors from GSE2990 (Sotiriou et al., 2006) and reported that while GSEA identifies only three cancer related pathways in both datasets, AF identifies four. (Table 1 in their paper). Furthermore, they reported one overlapping pathway in the top 5 results for GSEA, and two overlapping pathways in the top 5 results of AF (Figure 2b of their paper). We applied SIGORA to the same two datasets (DE genes for grade 1 *vs*. 3 samples were obtained from Genomic portals (Shinde et al., 2010) at a p-value of 0.001) and observed more consistent results than AF and GSEA. There were four common cancer related pathways within the top five results *("Cell cycle", "RNA transport", "Proteasome"* and  *"Spliceosome"*), and a fifth pathway (*"DNA replication"*) within the top seven results for the two datasets. Additional consistently significant pathways identified by SIGORA include "*Colorectal cancer*”, "*Oocyte meiosis*", "*Cysteine and methionine metabolism*", "*Aminoacyl-tRNA biosynthesis*" and "*Base excision repair*".

*Identification of ‘target pathways’ on a large collection of datasets.* The authors of PADOG (Tarca, Draghici, Bhatti, & Romero, 2012) have proposed a different, presumably objective criterion to measure the performance of their method: they selected 24 expression datasets where the corresponding disease is the name of a KEGG pathway. The KEGG pathway describing that disease was then considered to be the ‘target pathway’ for this dataset. The analysis methods were compared in terms of their ability to identify the target pathway as statistically significant in the analysis of each data set. They report that PADOG was able to identify one (4.2%) of the 24 target pathways as significant (after adjusting for multiple testing) whereas GSEA and GSA did not identify any of the target pathways. In order to compare SIGORA to PADOG using this benchmark, SIGORA was applied to the 24 lists of differentially expressed (p < 0.0001) genes from these 24 datasets. In two cases (GSE9348 and GSE9476), SIGORA identified the target pathway as significant after adjusting for multiple testing. This is twice the number of such hits by PADOG on these datasets.

**References for Suppl. Text 1**

Allali, O., Magnien, C., & Latapy, M. (2013). Internal link prediction : a new approach for predicting links in bipartite graphs. *Dynamic Networks and Knowledge Discovery, special issue of Intelligent Data Analysis*, *7*(1), 5–25.

Goeman, J. J., & Bühlmann, P. (2007). Analyzing gene expression data in terms of gene sets: methodological issues. *Bioinformatics (Oxford, England)*, *23*(8), 980–7. doi:10.1093/bioinformatics/btm051

Huang, D. W., Sherman, B. T., & Lempicki, R. a. (2009). Bioinformatics enrichment tools: paths toward the comprehensive functional analysis of large gene lists. *Nucleic acids research*, *37*(1), 1–13. doi:10.1093/nar/gkn923

Jiao, X., Sherman, B. T., Huang, D. W., Stephens, R., Baseler, M. W., Lane, H. C., & Lempicki, R. A. (2012). DAVID-WS: a stateful web service to facilitate gene/protein list analysis. *Bioinformatics (Oxford, England)*, *28*(13), 1805–6. doi:10.1093/bioinformatics/bts251

Lynn, D. J., Winsor, G. L., Chan, C., Richard, N., Laird, M. R., Barsky, A., Gardy, J. L., et al. (2008). InnateDB: facilitating systems-level analyses of the mammalian innate immune response. *Molecular systems biology*, *4*(218), 218. doi:10.1038/msb.2008.55

Ma, J., Sartor, M. a, & Jagadish, H. v. (2011). Appearance frequency modulated gene set enrichment testing. *BMC Bioinformatics*, *12*(1), 81. doi:10.1186/1471-2105-12-81

Miller, L. D., Smeds, J., George, J., Vega, V. B., Vergara, L., Ploner, A., Pawitan, Y., et al. (2005). An expression signature for p53 status in human breast cancer predicts mutation status, transcriptional effects, and patient survival. *Proceedings of the National Academy of Sciences of the United States of America*, *102*(38), 13550–5. doi:10.1073/pnas.0506230102

Obayashi, T., & Kinoshita, K. (2011). COXPRESdb: a database to compare gene coexpression in seven model animals. *Nucleic acids research*, *39*(Database issue), D1016–22. doi:10.1093/nar/gkq1147

Padrón, B., Nogales, M., & Traveset, A. (2011). Alternative approaches of transforming bimodal into unimodal mutualistic networks. The usefulness of preserving weighted information. *Basic and Applied Ecology*, *12*(8), 713–721. doi:10.1016/j.baae.2011.09.004

Reimand, J., Kull, M., Peterson, H., Hansen, J., & Vilo, J. (2007). g:Profiler--a web-based toolset for functional profiling of gene lists from large-scale experiments. *Nucleic acids research*, *35*(Web Server issue), W193–200. doi:10.1093/nar/gkm226

Shinde, K., Phatak, M., Johannes, F. M., Chen, J., Li, Q., Vineet, J. K., Hu, Z., et al. (2010). Genomics Portals: integrative web-platform for mining genomics data. *BMC genomics*, *11*(1), 27. doi:10.1186/1471-2164-11-27

Sotiriou, C., Wirapati, P., Loi, S., Harris, A., Fox, S., Smeds, J., Nordgren, H., et al. (2006). Gene expression profiling in breast cancer: understanding the molecular basis of histologic grade to improve prognosis. *Journal of the National Cancer Institute*, *98*(4), 262–72. doi:10.1093/jnci/djj052

Subramanian, A., Tamayo, P., Mootha, V. K., Mukherjee, S., Ebert, B. L., Gillette, M. A., Paulovich, A., et al. (2005). Gene set enrichment analysis: A knowledge-based approach for interpreting genome-wide expression profiles. *Proceedings of the National Academy of Sciences of the United States of America*, *102*(43), 15545–15550. Retrieved from http://www.pubmedcentral.nih.gov/articlerender.fcgi?artid=1239896&tool=pmcentrez&rendertype=abstract

Tarca, A. L., Draghici, S., Bhatti, G., & Romero, R. (2012). Down-weighting overlapping genes improves gene set analysis. *BMC bioinformatics*, *13*(1), 136. doi:10.1186/1471-2105-13-136
