# Supplementary figures and images for: Pathway-GPS and SIGORA: identifying relevant pathways based on the over-representation of their gene-pair signatures
Source: PeerJ. 2013 Dec 19;1:e229. doi: 10.7717/peerj.229 (PMC3883547; doi:10.7717/peerj.229)

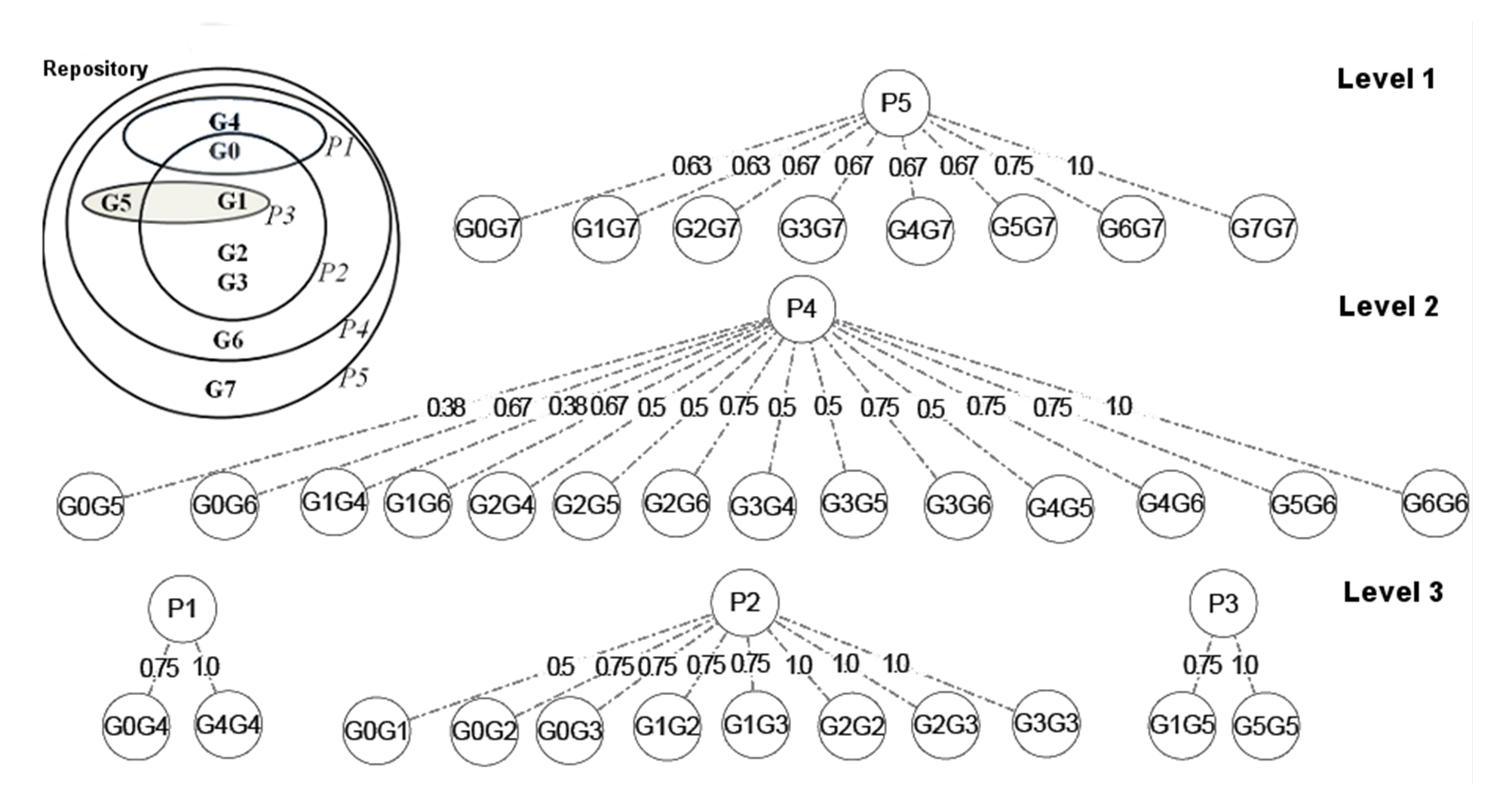

Supplement: Figure S1 — Here, G0 to G7 are genes that are annotated in the hierarchically organized pathways P1 to P5, as shown in the Vann-Diagram (inset). In the first iteration, only signatures for the outer-most level of the hierarchy(P5) are determined. The GPS for the higher levels are the ones associated with the less general pathways, and are only visible after removal of the more general terms. For example, the GPS of P1, P2 and P3 are only visible at level 3, after removal of P5 and P4. [file peerj-01-229-s002.png]
